# Increased Corrected Index of Cardiac Electrophysiological Balance in Behçet’s Disease: Association with Disease Duration

**DOI:** 10.3390/jcm15155775

**Published:** 2026-07-23

**Authors:** Aylin Dolu Karaca, Mehmet Cansel, Yücel Karaca, Şeyda Şahin, Çetin Mirzaoğlu, Mehdi Karasu, Ahmet Karataş, Mehmet Ali Kobat

**Affiliations:** 1 Department of Rheumatology, Adıyaman Training and Research Hospital, 02200 Adıyaman, Türkiye; nilyaulod@hotmail.com; 2 Department of Cardiology, Faculty of Medicine, Inonu University, 44210 Malatya, Türkiye; 3 Department of Cardiology, Adıyaman Training and Research Hospital, 02200 Adıyaman, Türkiye; yucel__karaca@hotmail.com; 4 Department of Cardiology, Elazığ Fethi Sekin City Hospital, 23280 Elazığ, Türkiye; seydashn.58@gmail.com (Ş.Ş.); dr.cmirzaoglu@gmail.com (Ç.M.); mehdikarasu@yahoo.com (M.K.); 5 Department of Rheumatology, Fırat University, 23119 Elazığ, Türkiye; drakaratas@yahoo.com; 6 Department of Cardiology, Fırat University, 23119 Elazığ, Türkiye; mkobat@firat.edu.tr

**Keywords:** Behçet’s disease, cardiac electrophysiology, index of cardiac electrophysiological balance, ventricular arrhythmia, QTc interval, inflammation

## Abstract

**Background/Objectives:** Behçet’s disease (BD) is a chronic multisystem inflammatory vasculitis associated with an increased risk of ventricular arrhythmias and sudden cardiac death. The index of cardiac electrophysiological balance (iCEB) and its corrected form (iCEBc) are novel electrocardiographic markers reflecting the balance between ventricular depolarization and repolarization and have been proposed as indicators of arrhythmic risk. This study aimed to evaluate iCEB and iCEBc in patients with BD and to investigate their associations with disease duration and inflammatory activity. **Methods:** This observational, cross-sectional study included 81 patients with clinically inactive BD (Behçet’s Disease Current Activity Form score 0–1) and 84 age- and sex-matched healthy controls. All participants underwent laboratory testing, transthoracic echocardiography and standard 12-lead electrocardiography. QT, QTc, QRS, iCEB (QT/QRS), and iCEBc (QTc/QRS) values were calculated. Correlation analyses were performed to evaluate the relationships between disease duration and QTc, iCEB, iCEBc, and CRP levels in the Behçet group. Disease duration, age, and CRP levels were subsequently entered into a multivariable linear regression model to identify independent predictors of iCEBc. **Results:** Patients with BD exhibited significantly higher QTc (431.41 ± 24.18 vs. 410.60 ± 18.65 ms, *p* < 0.001), iCEB (4.49 ± 0.33 vs. 4.36 ± 0.35, *p* = 0.016), and iCEBc (5.20 ± 0.47 vs. 4.93 ± 0.33, *p* < 0.001) values compared with healthy controls. CRP levels were also significantly elevated in the Behçet group (23.34 ± 34.05 vs. 6.07 ± 4.57 mg/L, *p* < 0.001). Disease duration demonstrated significant positive correlations with QTc (r = 0.539, *p* < 0.001), iCEB (r = 0.447, *p* < 0.001), iCEBc (r = 0.747, *p* < 0.001), and CRP levels (r = 0.419, *p* < 0.001). In multivariable linear regression analysis, disease duration emerged as the sole independent predictor of iCEBc (β = 0.077, 95% CI: 0.057–0.097, *p* < 0.001), whereas CRP was not independently associated with iCEBc (*p* = 0.286). **Conclusions:** Patients with BD have significantly increased iCEB and iCEBc values, indicating impaired cardiac electrophysiological balance. The strong association between disease duration and iCEBc suggests a relationship between disease duration and electrophysiological alterations associated with ventricular arrhythmia risk. iCEBc may serve as a simple and noninvasive electrocardiographic marker of electrophysiological alterations associated with ventricular arrhythmia risk in patients with BD.

## 1. Introduction

Behçet’s disease (BD) is a common and progressive vasculitis of unknown etiology, characterized by a chronic relapsing course with recurrent oral and genital ulcerations accompanied by ocular inflammation [1]. The disease usually begins in the third to fourth decades of life and is more frequently observed in populations along the historical Silk Road extending from East Asia to the Middle East and Mediterranean countries [2,3]. 

Cardiac involvement in BD is rare, also referred to as “Cardio-Behçet disease,” and mortality rates ranging from 7% to 46% have been reported in the literature [4,5]. Cardio-Behçet disease includes conditions such as pericarditis, myocarditis, endocarditis, dilated cardiomyopathy, right heart-related endomyocardial fibrosis, conduction system disorders, atrial fibrillation, life-threatening ventricular arrhythmias, and sudden cardiac death (SCD) [6,7]. Previous studies have shown that ventricular arrhythmias and SCD are more common in patients with BD [8,9]. However, the mechanism underlying the increased frequency of ventricular arrhythmias and SCD in BD has not yet been clearly elucidated.

The QT interval and corrected QT (QTc) reflect the periods of ventricular depolarization and repolarization and are influenced by multiple factors such as autonomic activity, genetic predisposition, acid–base disturbances, medications, underlying cardiac diseases, and electrolyte fluctuations [10]. For many years, electrocardiographic abnormalities in QT interval duration have been known to be associated with an increased risk of ventricular arrhythmias and sudden cardiac death [11]. However, conventional repolarization parameters may not fully reflect the complex interaction between ventricular depolarization and repolarization.

The index of cardiac electrophysiological balance (iCEB), calculated as the QT/QRS ratio, and the corrected iCEB (iCEBc), calculated as the QTc/QRS ratio, are novel and noninvasive markers developed to predict the occurrence of malignant ventricular arrhythmias [12]. While the QT interval represents a single cycle of ventricular contraction and relaxation, iCEB focuses on the balance between depolarization and repolarization of the action potential [13]. In addition, changes in iCEBc values have been reported to occur earlier and more prominently than corresponding changes in QTc [14,15]. Both increased and decreased ICEB values have been associated with ventricular arrhythmogenic risk; higher ICEB values are linked to torsades de pointes (TdP), whereas lower ICEB values are associated with ventricular tachycardia and ventricular fibrillation developing through non-TdP mechanisms [15,16].

Despite growing interest in iCEB and iCEBc, data regarding these parameters in patients with BD remain limited. Furthermore, the potential relationship between cardiac electrophysiological balance, disease duration, and inflammatory burden has not been adequately investigated. Chronic inflammation may contribute to myocardial electrical remodeling by altering ion channel expression, membrane potential, calcium handling, and gap junction function, thereby disrupting the electrophysiological properties of cardiomyocytes and increasing susceptibility to arrhythmias. Therefore, considering the chronic inflammatory nature of BD and its potential effects on myocardial electrical remodeling, evaluation of iCEB and iCEBc may provide valuable insights into arrhythmic risk stratification in this population.

Therefore, the present study aimed to evaluate iCEB and iCEBc values in patients with BD and to investigate their associations with disease duration and inflammatory activity.

## 2. Materials and Methods

### 2.1. Study Design

This observational, cross-sectional study was conducted at the Rheumatology and Cardiology outpatient clinics of Adıyaman Training and Research Hospital and Fırat University Hospital between 1 April 2023 and 1 January 2025.

### 2.2. Study Population

All electrocardiographic recordings and clinical evaluations were performed during a clinically inactive phase of Behçet’s disease. Clinical disease activity was assessed using the Behçet’s Disease Current Activity Form (BDCAF), and only patients with a BDCAF score of 0 or 1 were considered to have inactive disease and were eligible for inclusion in the study. A total of 95 patients diagnosed with Behçet’s disease according to the International Study Group criteria were initially screened for eligibility [17]. Fourteen patients were excluded for the following reasons: active disease (BDCAF score ≥ 2; *n* = 9), atrial fibrillation (*n* = 1), QRS duration ≥ 120 ms (*n* = 2), left ventricular dysfunction secondary to coronary artery disease (*n* = 1), moderate mitral regurgitation (*n* = 1), previous myocardial infarction (*n* = 2), beta-blocker use (*n* = 3), neoplastic disease (lung cancer; *n* = 1), moderate renal insufficiency (estimated glomerular filtration rate < 60 mL/min/1.73 m^2^; *n* = 1), and refusal to participate (*n* = 1). Because some patients met more than one exclusion criterion, the total number of exclusion reasons exceeded the number of excluded patients. The final analysis included 81 patients with BD. The control group initially consisted of 87 age- and sex-matched healthy volunteers. Three volunteers were excluded because of QRS duration ≥ 120 ms (*n* = 1), moderate mitral regurgitation (*n* = 1), and beta-blocker use for hypertension (*n* = 1). Consequently, the final control group comprised 84 healthy controls.

### 2.3. Exclusion Criteria

Patients with hepatic or renal insufficiency, neoplastic disease, left ventricular dysfunction, moderate or severe valvular heart disease or a mechanical prosthetic valve, a history of pacemaker or cardioverter-defibrillator implantation, atrial fibrillation or atrial flutter, inadequate image quality on two-dimensional echocardiography, thyroid dysfunction, QRS duration ≥ 120 ms, or bundle branch block were excluded from the study. All participants were in sinus rhythm and had not used antiarrhythmic drugs, beta-blockers, non-dihydropyridine calcium channel blockers, tricyclic antidepressants, antihistamines, or antipsychotics for at least 6 months prior to the study.

### 2.4. Clinical Evaluation

Demographic characteristics (age and sex) were recorded for all participants. The presence of hypertension and diabetes mellitus was documented. Disease duration was determined by defining the onset as the date on which the patient first fulfilled all International Study Group criteria for BD and was expressed in years.

### 2.5. Laboratory Investigations

Venous blood samples were obtained from all participants after at least 8 h of fasting. Measurements included fasting blood glucose, creatinine, total cholesterol, triglycerides, high-density lipoprotein (HDL-C), low-density lipoprotein (LDL-C), sodium, potassium, calcium, magnesium, hemoglobin, white blood cell (WBC) count, neutrophil count, lymphocyte count, platelet count, and CRP levels.

### 2.6. Electrocardiographic and Echocardiographic Assessments

Echocardiographic examinations were performed in the left lateral decubitus position using a Vivid 8 ultrasound system (GE Healthcare, Horten, Norway). Apical four- and two-chamber views, and left ventricular ejection fraction was calculated using Simpson’s biplane method.

For electrocardiography, an ECG recorder (Nihon Kohden Corporation, Tokyo, Japan) operating at a paper speed of 25 mm/s and a voltage of 10 mm/mV was used. Resting recordings were obtained in the supine position. Each ECG tracing was scanned and magnified by 400% using Adobe Photoshop 2024 (Adobe Inc., San Jose, CA, USA). ECGs were evaluated by two cardiologists (Ç.M. and M.K.) blinded to group allocation. Heart rate (beats/min), QRS duration, and QT interval (measured in leads V5 and DII) were recorded. The QTc interval was corrected using Bazett’s formula (QT/√RR). iCEB was calculated as the QT/QRS ratio, and iCEBc was calculated as the QTc/QRS ratio [15,16].

### 2.7. Statement of Ethics

The study protocol was approved by the Ethics Committee of Fırat University (Decision dated 16 March 2023, number 15136), and the study was conducted in accordance with the ethical principles of the Declaration of Helsinki. Written informed consent was obtained from all participants.

### 2.8. Statistical Analysis

Statistical analyses were performed using IBM SPSS Statistics for Windows, Version 22.0 (IBM Corp., Armonk, NY, USA). Data distribution was assessed using the Kolmogorov-Smirnov test. Continuous variables are presented as mean ± standard deviation or median (interquartile range), as appropriate. Categorical variables are presented as frequencies and percentages.

Comparisons between groups were performed using the independent samples *t*-test, Mann–Whitney U test, chi-square test, or Fisher’s exact test, as appropriate. Correlation analyses were performed to evaluate the relationships between disease duration and QTc, iCEB, iCEBc, and CRP levels in the Behçet group using Pearson’s or Spearman’s correlation coefficients.

Disease duration, CRP level, and age were entered into a multivariable linear regression model to identify independent predictors of iCEBc. QTc and iCEB were not included in the regression model because of their mathematical relationship with iCEBc and the potential risk of collinearity. Therefore, only variables that were not mathematically derived from iCEBc and were considered clinically relevant were included in the multivariable regression model. A two-sided *p* value < 0.05 was considered statistically significant.

## 3. Results

A total of 165 participants were included in the analysis, consisting of 81 patients with clinically inactive BD and 84 age- and sex-matched healthy controls. The demographic, laboratory, echocardiographic, and electrocardiographic characteristics of the study population are summarized in Table 1.

There were no statistically significant differences between the groups with respect to age or female sex distribution. Diabetes mellitus was present in 9 patients (11.1%) in the Behçet group and 11 subjects (13.1%) in the control group. Hypertension was observed in 12 patients (14.8%) with BD and in 10 controls (11.9%). Among laboratory parameters, CRP levels were significantly higher in the Behçet group compared with the control group. No statistically significant differences were observed between the groups in glucose, creatinine, total cholesterol, LDL-C, triglycerides, HDL-C, sodium, potassium, calcium, magnesium, hemoglobin, WBC count, neutrophil count, lymphocyte count, or platelet count (Table 1).

Echocardiographic and electrocardiographic evaluation demonstrated no significant differences between groups in left ventricular ejection fraction, heart rate, QRS duration, or QT interval. In contrast, the QTc interval was significantly longer in patients with BD. In addition, both iCEB and iCEBc were significantly higher in the Behçet group (Table 1).

The median disease duration in patients with BD was 6 years (interquartile range: 4–9 years). Correlation analysis demonstrated significant positive associations between disease duration and QTc interval, iCEB, iCEBc, and CRP levels (Table 2).

**Figure 1:**
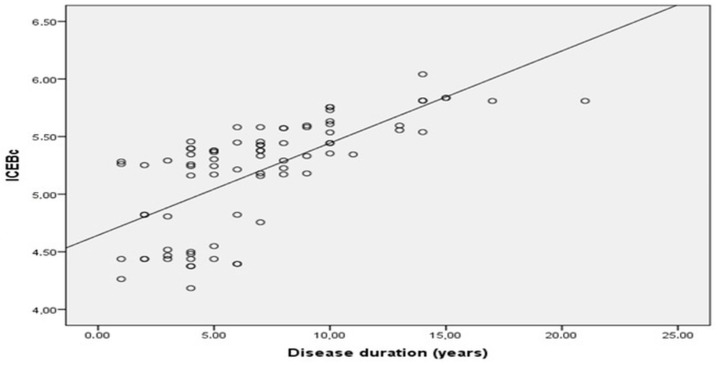
Relationship between disease duration and ICEBc in patients with Behçet’s disease.

To determine the independent predictors of iCEBc, a multivariable linear regression analysis was performed using age, disease duration, and CRP level as covariates. Disease duration remained the sole independent predictor of iCEBc, whereas neither age nor CRP level was independently associated with iCEBc (Table 3). The relationship between disease duration and iCEBc is illustrated in Figure 1, demonstrating a strong positive association between increasing disease duration and higher iCEBc values.

## 4. Discussion

The present study investigated cardiac electrophysiological balance in patients with BD and demonstrated several important findings. First, QTc, iCEB, and iCEBc values were significantly higher in patients with BD than in healthy controls. Second, disease duration was positively correlated with QTc, iCEB, iCEBc, and CRP levels. Third, disease duration emerged as the sole independent predictor of iCEBc in multivariable regression analysis. These findings suggest that longer disease duration may be associated with progressive electrophysiological alterations associated with ventricular arrhythmia risk in patients with BD. Although increased iCEBc values may reflect electrophysiological alterations associated with ventricular arrhythmia risk, clinical arrhythmic outcomes were not evaluated in the present study; therefore, no direct conclusions can be drawn regarding ventricular arrhythmic susceptibility. Accordingly, our findings should be interpreted as evidence of electrophysiological alterations associated with ventricular arrhythmia risk rather than confirmed increased ventricular arrhythmic risk.

Although cardiac involvement is relatively uncommon in BD, it is associated with substantial morbidity and mortality. Previous studies have reported that ventricular arrhythmias and sudden cardiac death occur more frequently in patients with BD than in the general population [8,9]. Furthermore, studies using advanced imaging techniques and myocardial deformation analyses have demonstrated clinically silent structural and functional myocardial injury in patients with BD [18,19]. Consistent with these observations, our study demonstrated significant alterations in electrocardiographic parameters associated with ventricular arrhythmogenesis.

The QT and QTc intervals are established parameters that have long been used to evaluate ventricular arrhythmic risk [20]. However, when considered alone, these parameters may not adequately reflect the balance between ventricular depolarization and repolarization [15]. iCEB, and particularly iCEBc, are novel electrocardiographic markers that assess these two processes simultaneously. Previous studies have suggested that iCEBc may reflect arrhythmogenic risk earlier and more prominently than QTc [13,15,16]. In our study, both iCEB and iCEBc values were significantly higher in patients with BD. Furthermore, among all evaluated electrocardiographic parameters, iCEBc demonstrated the strongest correlation with disease duration, and disease duration emerged as the sole independent predictor of iCEBc in multivariable analysis. However, because of the cross-sectional design of the present study, we were unable to determine whether iCEBc changes earlier than QTc during the course of Behçet’s disease. Therefore, our findings indicate that iCEBc showed a stronger association with disease duration than QTc, rather than demonstrating earlier temporal changes or superior sensitivity.

One of the most important findings of the present study was the strong positive correlation between disease duration and iCEBc. Furthermore, disease duration remained independently associated with iCEBc after multivariable adjustment. This observation suggests that longer disease duration may be associated with progressive electrophysiological alterations beyond those reflected by a single measurement of inflammatory activity. Long-term exposure to systemic inflammation may promote structural and electrophysiological changes within the myocardium, resulting in progressive alterations in ventricular depolarization–repolarization balance. Importantly, all patients included in the present study had clinically inactive disease (BDCAF score 0–1). Therefore, these findings suggest that myocardial electrical remodeling may persist even during periods of clinical remission, possibly reflecting the cumulative effects of chronic inflammation rather than active disease alone.

Although CRP levels were significantly elevated in patients with BD and showed a positive correlation with disease duration, CRP was not independently associated with iCEBc in multivariable analysis. In the present study, CRP was included as an objective biomarker of systemic inflammation rather than as a surrogate measure of clinical disease activity. Because all enrolled patients had clinically inactive disease according to BDCAF (score 0–1), the observed relationship between CRP and iCEBc most likely reflects variability in residual systemic inflammatory burden during clinical remission rather than differences in overt disease activity.

From a clinical perspective, iCEBc appears to be a simple, inexpensive, and noninvasive electrocardiographic marker that can be obtained from a standard 12-lead ECG. The observed association between prolonged disease duration and increased iCEBc values suggests that closer cardiac monitoring may be warranted in patients with longstanding BD, even in the absence of overt cardiovascular involvement.

In conclusion, our findings indicate that patients with BD exhibit increased iCEB and iCEBc values. The strong relationship between disease duration and iCEBc suggests that longer disease duration is associated with progressive electrophysiological alterations. These findings support an association between Behçet’s disease and altered electrocardiographic markers and warrant further prospective studies to determine their clinical significance.

## 5. Limitations

This study has several limitations. First, due to its cross-sectional design, a causal relationship between disease duration and alterations in cardiac electrophysiological balance cannot be established; the findings reflect associations only. Second, Holter ECG monitoring and long-term clinical arrhythmic follow-up were not performed. Therefore, the prognostic significance of increased iCEB and iCEBc values for clinically relevant ventricular arrhythmias could not be determined. Third, the study was conducted at a single center with a relatively limited sample size, which may restrict the generalizability of the findings. Fourth, advanced imaging modalities such as cardiac magnetic resonance imaging were not performed to evaluate subclinical myocardial involvement. Furthermore, cumulative disease damage was not assessed using a validated damage assessment instrument such as the Behçet’s Syndrome Overall Damage Index (BODI). Therefore, although disease duration may partially reflect cumulative disease burden, it should not be considered a comprehensive measure of cumulative disease damage. Because only patients with clinically inactive Behçet’s disease (BDCAF score 0–1) were included, the relationship between iCEBc and different levels of clinical disease activity could not be evaluated. Future studies including patients with both active and inactive disease are needed to determine whether iCEBc varies according to disease activity. Moreover, detailed longitudinal treatment characteristics, including cumulative exposure to colchicine, corticosteroids, immunosuppressive agents, and biologic therapies, were not systematically collected. Because treatment regimens in Behçet’s disease may change substantially over time, treatment status at a single cross-sectional assessment would not accurately reflect cumulative treatment exposure. Therefore, the potential influence of long-term treatment history on inflammatory burden and electrophysiological parameters could not be evaluated. Finally, only a single resting electrocardiogram was evaluated, and temporal variability in electrocardiographic parameters could not be taken into account.

Despite these limitations, this study is among the few investigations evaluating cardiac electrophysiological balance in patients with BD and, to our knowledge, is the first to demonstrate a strong association between disease duration and iCEBc. These findings provide novel insights into the potential relationship between cumulative disease burden and ventricular electrical remodeling in BD.

## Figures and Tables

**Table 1:** Demographic, laboratory, echocardiographic, and electrocardiographic characteristics of the study population.

	Behçet’s Disease (*n* = 81)	Controls (*n* = 84)	*p*
Age (y)	42.46 ± 10.53	39.54 ± 10.73	0.079
Female sex, *n* (%)	41 (50.6%)	40 (47.6%)	0.819
Diabetes mellitus, *n* (%)	9 (11.1)	11 (13.1)	0.879
Hypertension, *n* (%)	12 (14.8)	10 (11.9)	0.748
Glucose (mg/dL)	114.32 ± 46.58	107.38 ± 34.99	0.282
Creatinine (mg/dL)	0.83 ± 0.16	0.85 ± 0.16	0.408
Total cholesterol (mg/dL)	183.95 ± 42.52	192.35 ± 40.39	0.196
LDL-C (mg/dL)	120.86 ± 41.31	119.95 ± 39.52	0.885
Triglyceride (mg/dL)	140.11 ± 74.83	148.32 ± 76.90	0.488
HDL-C (mg/dL)	43.15 ± 11.61	45.63 ± 10.45	0.151
Sodium (mmol/L)	139.17 ± 2.49	138.54 ± 2.49	0.103
Potassium (mmol/L)	4.15 ± 0.47	4.08 ± 0.55	0.408
Calcium (mg/dL)	9.03 ± 0.30	9.01 ± 0.30	0.595
Magnesium (mg/dL)	1.93 ± 0.25	1.97 ± 0.25	0.293
Hemoglobin (g/dL)	14.02 ± 1.70	13.95 ± 1.70	0.796
WBC (×10^9^/L)	10.85 ± 4.29	10.52 ± 3.17	0.580
Neutrophils (×10^9^/L)	8.02 ± 4.08	7.18 ± 3.15	0.142
Lymphocytes (×10^9^/L)	2.05 ± 1.17	2.28 ± 1.21	0.219
Platelets (×10^9^/L)	239.00 ± 80.83	261.85 ± 88.18	0.084
CRP (mg/L)	23.34 ± 34.05	6.07 ± 4.57	<0.001
Ejection fraction (%)	61.86 ± 3.46	62.55 ± 2.84	0.169
Heart rate (beats/min)	77.72 ± 10.08	79.07 ± 10.29	0.394
QRS duration (ms)	82.30 ± 4.37	83.36 ± 4.26	0.116
QT interval (ms)	368.54 ± 24.59	362.52 ± 23.81	0.112
QTc interval (ms)	431.41 ± 24.18	410.60 ± 18.65	<0.001
ICEB	4.49 ± 0.33	4.36 ± 0.35	0.016
ICEBc	5.20 ± 0.47	4.93 ± 0.33	<0.001
Disease duration (years) *	6 (4–9)	—	—

Abbreviations: CRP, C-reactive protein; HDL-C, high-density lipoprotein cholesterol; ICEB, index of cardiac electrophysiological balance (QT/QRS); ICEBc, corrected index of cardiac electrophysiological balance (QTc/QRS); LDL-C, low-density lipoprotein cholesterol; QTc, corrected QT interval; WBC, white blood cell count. * Disease duration is presented as median (interquartile range) and applies only to the Behçet group.

**Table 2:** Correlation of disease duration with significant parameters in Behçet’s disease.

	Disease Duration	
	r	*p*
QTc (ms)	0.539	<0.001
ICEB	0.447	<0.001
ICEBc	0.747	<0.001
CRP (mg/dL)	0.419	<0.001

Abbreviations: CRP, C-reactive protein; ICEB, index of cardiac electrophysiological balance; ICEBc, corrected index of cardiac electrophysiological balance; QTc, corrected QT interval.

**Table 3:** Multivariable linear regression analysis of independent predictors of the corrected index of cardiac electrophysiological balance (ICEBc) in patients with Behçet’s disease.

	ICEBc	
Variables	β (95% CI)	*p*
CRP (mg/dL)	0.001 (−0.001–0.004)	0.277
Disease duration (years)	0.076 (0.054–0.098)	<0.001
Age (years)	0.001 (−0.008–0.009)	0.881

Abbreviations: ICEBc, corrected index of cardiac electrophysiological balance; CRP, C-reactive protein; CI, confidence interval.

## Data Availability

The data supporting the findings of this study are included within the article. Additional information is available from the corresponding author upon reasonable request.

## Abbreviations

The following abbreviations are used in this manuscript:

BD	Behçet’s disease
iCEB	Index of cardiac electrophysiological balance
iCEBc	Corrected index of cardiac electrophysiological balance
CRP	C-reactive protein
SCD	Sudden cardiac death
HDL-C	High-density lipoprotein
LDL-C	Low-density lipoprotein
WBC	White blood cell
QTc	Corrected QT interval
